# Microbes in the Water Infrastructure: Underpinning Our Society

**DOI:** 10.1264/jsme2.ME3102rh

**Published:** 2016-06

**Authors:** Takashi Narihiro

**Affiliations:** 1 Bioproduction Research Institute, National Institute of Advanced Industrial Science and Technology (AIST)Tsukuba Central 6, Tsukuba, Ibaraki 305–8566Japan

How do you feel when you hear the words “wastewater,” “sludge,” “sewer,” and “sewage”? These words may make you feel uncomfortable. However, as Victor Hugo wrote in “Les Misérables,” these words are inevitably related to human society (16):

“... *The history of men is reflected in the history of sewers. The Germoniae narrated Rome. The sewer of Paris has been an ancient and formidable thing. It has been a sepulchre, it has served as an asylum. Crime, intelligence, social protest, liberty of conscience, thought, theft, all that human laws persecute or have persecuted, is hidden in that hole* ...”

Since ancient Roman times, sewer pipe/tunnel networks have been built beneath urban areas to gather sewage/wastewater discharged from human activity (9). Biological treatment technologies such as activated sludge (3–5) and anaerobic digestion (AD) (6, 41) have been developed since the early 1900s to remediate sewage/wastewater constituents. Nowadays, these biological treatment processes are used as the core technology of modern municipal wastewater treatment processes (WWTP) and have become the underlying infrastructure for society. Within these processes, diverse microorganisms (eukaryotes [15, 33] and prokaryotes [35, 56]) form “sludge” and play a critical role in sewage/wastewater remediation. Thus, the characterization of sludge microbial assemblages and identification of their *in situ* functions have been conducted using cultivation, microscopic, and molecular techniques.

Nitrification, the stepwise biological conversion of ammonia to nitrate, is an important step in municipal WWTP. The long-term (*i.e.*, between 5 and 6 years) microbial community monitoring of activated sludge systems by employing the modern massive parallel sequencing of 16S rRNA gene amplicons revealed the temporal dynamics of core community members including nitrifying bacteria (*i.e.*, *Nitrosomonas*, *Nitrospira*, and “*Ca.* Nitrotoga”) (20, 51). Microradiography combined with fluorescent *in situ* hybridization (MAR-FISH) showed that “*Ca.* Nitrotoga”-type nitrite-oxidizing bacteria (NOB) may play a role in nitrite-dependent autotrophic carbon fixation (30). Using the β-subunit of the nitrite oxidoreductase (*nxrB*) gene, amplicon pyrosequencing uncovered the high diversity of *Nitrospira* spp., expanding to lineages I, II, and the novel ‘WWTP Ingolstadt 454 lineage’, in activated sludge systems (13). Fujitani and colleagues (12) enriched *Nitrospira*-type NOB from nitrifying sludge by using a laboratory-scale continuous feeding reactor with porous polyester non-woven fabric materials as biomass carriers. They then successfully isolated the novel bacterial species “*Nitrospira japonica*,” associated with *Nitrospira* lineage II (54). Itoh *et al.* (19) isolated the thermotolerant ammonia oxidizer *Nitrosomonas* sp. JPCCT2, which is capable of growing at temperatures of up to 48°C, from activated sludge in a thermal power station. Until recently, nitrification was considered to be performed by two metabolically distinct organisms, *i.e.*, an ammonia oxidizer and nitrite oxidizer. As if to rewrite the textbook of microbiology, Daims *et al.* (10) isolated “*Ca.* Nitrospira inopinata” as the first complete ammonia oxidizer (comammox) from a microbial biofilm developing on the walls of a pipe under a flow of hot water. In addition to this isolated representative, they identified several comammox-like metagenomic bins derived from a full-scale activated sludge tank, membrane bioreactor, and groundwater wells. Hanada *et al.* (14) also reported that organisms assigned to uncultivated phylum TM7 (“*Ca.* Saccharibacteria”) predominated in laboratory-scale acidophilic nitrifying sequencing-batch reactors (ANSBR), suggesting that TM7 plays a specific role in nitrification in acidic environments. Kindaichi *et al.* (24) very recently reported that “*Ca.* Saccharibacteria” organisms are capable of utilizing glucose under aerobic, anoxic (nitrate-reducing), and anaerobic conditions, as demonstrated using MAR-FISH and enzyme-labeled fluorescence combined with FISH (ELF-FISH) analyses.

An enhanced biological phosphorous removal (EBPR) system is implemented in many municipal WWTP to decrease phosphate concentrations in sewage/wastewater (11, 42). In the EBPR system, polyphosphate-accumulating organisms (PAO) intracellularly synthesize polyphosphate under aerobic conditions and utilize it as an energy resource for polyhydroxyalkanoates (PHA) and/or glycogen biosynthesis from carbon substrates under anaerobic conditions. Glycogen-accumulating organisms (GAO), competitors of PAO, accumulate PHA and glycogen and become predominant in deteriorated EBPR systems. The metabolic capacity and physiological traits of PAO (“*Ca.* Accumulibacter” [2], *Tetrasphaera*-type PAO [26], and *Microlunatus phosphovorus* strain NM-1 [23]) and GAO (“*Ca.* Competibacter”-type GAO [34] and “*Ca.* Defluviicoccus tetraformis” strain TFO71 [43]) have hitherto been characterized by (meta) genomic analyses. A 16S rRNA gene pyrotag-based microbial community analysis of a laboratory-scale EBPR reactor was meticulously conducted to investigate the dynamics of “Ca. Accumulibacter”-related PAO (50). Lv *et al.* (32) reported that *Dechloromonas*-related PAO outcompeted “*Ca.* Accumulibacter” in a laboratory-scale sequencing batch EBPR system operated under anaerobic-anoxic (denitrifying) conditions.

AD technology is widely used to decompose waste activated sludge (WAS; excess sludge generated from an activated sludge process) and remediate industrial wastewater (28, 39, 55). In the AD process, three microbial trophic assemblages basically forge a collaborative relationship to convert organic compounds in WAS/wastewater to CH_4_ and CO_2_: ‘fermenters’ degrade high-molecular-weight ingredients (*e.g.*, carbohydrates, proteins, and lipids) to volatile fatty acids (VFAs) and H_2_; syntrophic substrate oxidizers (‘syntrophs’) degrade VFAs to acetate and H_2_; and methanogenic archaea (‘methanogens’) further utilize acetate and H_2_ with the production of CH_4_ and CO_2_ (52). 16S rRNA gene-based microbial community profiling previously revealed that AD sludge embraced a large proportion of uncultivated organisms with unidentified ecophysiologies in AD (1, 7, 8, 27, 35, 36, 38, 45, 49, 53, 57). Recent omics-based analyses have provided a deeper insight into how uncultivated AD organisms function and survive. For example, members of the candidate phylum Hyd24-12 (“*Ca.* Fermentibacteria”), which is commonly observed in AD sludge, were metagenomically identified as sugar fermenters (25). The genomics of *Syntrophorhabdus aromaticivorans* strain UI, isolated from methanogenic sludge treating terephthalate wastewater, revealed its metabolic and energy conservation systems for the syntrophic degradation of aromatic compounds (44, 46). The *in situ* ecophysiology and synergistic interactions of uncultivated organisms including the candidate phyla “*Ca.* Atribacteria (OP9)”, “*Ca.* Cloacimonetes (WWE1)”, “*Ca.* Hydrogenedentes (NKB19)”, and “*Ca.* Marinimicrobia (SAR406)” in a methanogenic bioreactor treating terephthalate wastewater were investigated using a combined approach employing metagenomics, metatranscriptomics, and single-cell amplified genomics (45). In addition, Nobu *et al.* (47) successfully reconstructed four metagenomic bins of *Euryarchaeota* clade WSA2 (or ArcI), which is considered to play a role in methanogenesis in the AD process (7), and identified WSA2 as the first methanogen employing the demethylation of methylated thiols for methanogenesis. By considering its metabolic novelty and performing a genome-wide phylogenetic comparison between known *Euryarchaeota* organisms, they proposed the novel taxa “*Ca.* Methanofastidiosum methylthiophilus” of the class “*Ca.* Methanofastidiosa” for the WSA2 lineage. Iino *et al.* (17) also proposed the family *Methanomassiliicoccaceae* and order *Methanomassiliicoccales* with the *Methanomassiliicoccus*-related methanogen “*Ca.* Methanogranum caenicola” enriched from AD sludge.

Besides WWTP, concerted efforts have been made in an attempt to elucidate the microbial community structure of biofilms that develop in drinking water systems because it is important to ensure water quality and prevent health risks. A microbial community survey of water meters/pipes installed in the city of Landskrona, Sweden, showed that biofilms dominated by *Nitrospira*- and *Pedomicrobium*-related organisms developed in a water meter, in which red water (containing relatively high concentrations of iron and manganese) had been reported, while *Sphingomonadaceae-*proliferated biofilms were observed in other water meters without any issues (31). Karwautz and Lueders (22) investigated the effects of high-pressure hydraulic purging on biofilm microbial community structures in drinking water wells. They found that a significant decrease in the populations of *Pseudomonas* and *Legionellaceae*, which are recognized as potential pathogens of drinking water systems, was observed after the purging event. Ling and Liu (29) reported that a chloramination treatment may affect the dominant bacteria in artificially-developed biofilms in laboratory-scale reactors fed groundwater, and members of *Mycobacterium* that potentially harbor resistance to disinfectants through mycolic acid-containing cell walls may survive or grow under chloramination. Inoue *et al.* (18) found phylogenetically novel, functionally uncharacterized *Legionella*-associated clades from cooling tower/bath water samples using a clone library analysis combined with a DNA-intercalator ethidium monoazide treatment.

As discussed above, omics approaches may contribute to our understanding of the role of microbes in WWTP ecosystems, while cultivation/enrichment/microscopic approaches may facilitate the accuracy and robustness of omics-derived implications (21, 37, 40, 48). In “Les Misérables,” Jean Valjean carries wounded Marius on his shoulder through a long and difficult route in the Paris sewer system in order to save his life, and this leads to an outcome that makes Cosette and, perhaps, Jean Valjean himself happy. Unraveling the complete picture of the ecology and physiology of and interactions between microbes in the water infrastructure is also challenging. The happy ending of this story has not yet been reached.
